# The effects of acute responsive high frequency stimulation of the subiculum on the intra-hippocampal kainic acid seizure model in rats

**DOI:** 10.1002/brb3.70

**Published:** 2012-07-10

**Authors:** L Huang, G Luijtelaar

**Affiliations:** Department of Biological Psychology, Donders Center for Cognition, Donders Institute for Brain Cognition and Behaviour, Radboud University NijmegenNijmegen, The Netherlands

**Keywords:** High frequency stimulation, responsive, stimulation, subiculum, temporal lobe epilepsy

## Abstract

The effects of acute responsive high frequency stimulation (HFS) to the subiculum on seizures and interictal spikes were investigated in a semi-acute kainic acid (KA) induced seizure model in rats. Wistar rats (*n* = 15) were implanted with an electrode-cannula complex in the CA3 area, stimulation and recording electrodes in the subiculum and another recording electrode at the contralateral motor cortex. Two weeks later rats were injected repeatedly with KA (0.05 μg/0.1 μL) for 3 days with an interval of 48 h. HFS (125 Hz, 100 μsec) was delivered to the subiculum at a predetermined intensity range (100–500 μA) in the HFS group (*n* = 7) when seizures were visually detected, while no stimulation was delivered in the sham control group (*n* = 8). Various severities of seizures were obtained (Stage I–V) and all rats of both groups reached Stage V (Racine's scale) on Day 1. The HFS group had less focal seizures and a longer inter-focal seizure interval on Day 1. Interictal spike rate was also lower in the HFS group and decreased with injection days. Significant day effects were found for the latency, number of focal seizures, and duration of focal seizures and generalized seizures while differences between groups were no longer present. Responsive HFS did not disrupt ongoing seizures. However, focal seizures and interictal spikes were suppressed by HFS. Such anticonvulsant effects of acute subicular stimulation indicate that the subiculum is involved in seizure generation. The reduction of seizure sensitivity over the injection day reflects an intrinsic anticonvulsant mechanism.

## Introduction

Electrical stimulation of deep structures in the brain has been used successfully for treatment of movement disorders, such as Parkinson's disease. Recently, deep brain stimulation (DBS) has gained more attention as an alternative treatment for refractory epilepsy such as temporal lobe epilepsy (TLE). DBS has advantages as a reversible, less invasive treatment with fewer complications compared to temporal lobectomy; it offers a promising option for patients who are not eligible candidates for resective surgery.

Extensive evidence has shown that the hippocampus is pivotal in seizure generation (Swanson 1995; Spencer 2002). DBS, especially high frequency stimulation (HFS), has been applied to the hippocampus to control seizures in patients (Velasco et al. 2000a,b, 2001, 2007; Vonck et al. 2002; Osorio et al. 2005; Tellez-Zenteno et al. 2006; Boon et al. 2007) and in animal models (Bragin et al. 2002; Cuellar-Herrera et al. 2006; Wyckhuys et al. 2007, 2010a). The rational for HFS is that it is associated with desynchronization of neuronal activities and thereby might achieve therapeutic effects (Boon et al. 2007). Stimulation can be delivered at a predefined stimulation protocol, that is, scheduled stimulation, independent of the neurophysiological state of the brain. In contrast, responsive stimulation is delivered directly in response to electrographic epileptic activities. Considering that the occurrence of seizures can be irregular and intermittent, responsive stimulation has the potential advantages of targeting seizure dynamics with high temporal and spatial specificity and is less likely to cause tissue damage due to exposure of neuronal tissue to stimulation (Sunderam et al. 2010). Experimental studies indeed showed a reduction of spontaneous seizures by delivering high frequency responsive stimulation to the epileptogenic zone or to the anterior nucleus of thalamus in each four patients with inoperable TLE (Osorio et al. 2005). Additionally, afterdischarges were terminated or shortened by responsive brief bursts of pulse stimulation (Lesser et al. 1999; Motamedi et al. 2002) and seizures were altered or suppressed by responsive cortical stimulation in patients (Kossoff et al. 2004). Recently, implantable responsive neurostimulator (RNS; NeuroPace, Inc., Mountain View, CA) has been developed to detect real-time seizures and deliver responsive stimulation to patients with medically intractable partial-onset epilepsy. The safety and efficacy of this system has been assessed in a multicenter, double-blinded, randomized study in adults with medically refractory epilepsy (Morrell 2011). High frequency hippocampal stimulation was delivered during the preictal period on predicted spontaneous seizures in the status epilepticus (SE) rat model (Nair et al. 2006). Their preliminary results in three rats showed reduced seizure frequency and longer free seizure periods, indicating anticonvulsant effects of acute preictal hippocampal stimulation.

In the present study, acute responsive stimulation was delivered to one subregion of the hippocampus – the subiculum – in kainic acid (KA) treated rats. The subiculum is the major output structure in the hippocampal network (Witter and Groenewegen 1990; O'Mara et al. 2001), receiving fibers mainly from the CA1 field and projecting to the entorhinal cortex (EC), other cortical and subcortical structures (O'Mara 2005). Spontaneous rhythmic activity has been found in the isolated subiculum in human slices (Cohen et al. 2002; Wozny et al. 2003). It was also found that the subiculum was hyperexcitable when activated by CA1 or EC inputs in brain slices of pilocarpine treated rats (de Guzman et al. 2006). Taken together, the subiculum is rather prone to synchronous activities and has never been studied in the effects of responsive stimulation or scheduled stimulation to control seizures.

The aim of the study was to investigate the effects of responsive subicular HFS on temporal lobe seizures. A semi-acute temporal lobe seizure model was used: repeated injections of low dose KA intrahippocampally. With this seizure model not only different severities of seizures can be obtained but also a large number of seizures within a limited period. The presence of multiple focal and generalized seizures within a limited time frame provide us with multiple possibilities to intervene with responsive stimulation. The effects of responsive subicular stimulation were compared with a sham group. It is hypothesized that acute responsive HFS of the subiculum would interrupt seizures or reduce the rate of seizures and interictal spikes.

## Materials and Methods

### Animals

Male Wistar rats (*n* = 20), weighing 451 ± 47 g, were used (bred at the Biological Psychology Department, Radboud University Nijmegen). The rats were housed under controlled temperature (20°C, relative humidity 50–70%) and light conditions (12 h light/dark cycle with lights on at 8:00 A.M.), with ad libitum access to food and water. The local medical-ethical committee of the Radboud University Nijmegen (RU-DEC) approved all procedures on animal experimentation in this study. Efforts were taken to alleviate discomfort and number of animals in the study as much as possible.

### Simultaneous electrode-guide combinations

Simultaneous electrode-guide combinations (C315G-MS303/2; Plastics One, Roanoke, VA) comprised of a 26-gauge guide cannula and two insulated stainless steel wires glued to the guide cannula. This electrode-guide complex enables us to deliver KA into the injection site, record and stimulate very near the injection site. A dummy was used to close the guide cannula. The tips of bipolar electrodes were 1 mm shorter than the tip of cannula.

### Surgery

The rats were anesthetized with isoflurane inhalation and fixed in a stereotaxic frame. At the start of surgery atropine sulfate was given to reduce saliva secretion (0.1 mL, i.m.) and the analgesic Rimadyl (4 mg/kg, s.c.) was administered. Body temperature was monitored and maintained at 37°C with a heating pad throughout surgery. The cannula-bipolar electrode complex was placed in the CA3 area (AP: −5.6 mm, ML: −4.8 mm, DV: 5.0 mm). One tripolar electrode (MS222/2a; Plastics One) containing three stainless wires, was located on the left hemisphere, with the frontal wire targeting the motor cortex and the other two wires that were located above the cerebellum serving as reference and ground electrode. The other tripolar electrode was located in the subiculum (AP: −5.6 mm; ML: −2.2 mm; D: 3.2 mm) serving one recording and two stimulation electrodes. The cannula-electrode complex, tripolar electrodes, and several screws were attached to the skull with dental acrylic cement. After surgery, the animals were housed individually and were allowed to recover from surgery for 2 weeks. After that, the animals were handled by the experimenter 5 min per day.

### Video and EEG monitoring and stimulation set up

The rats were connected to the recording and stimulation leads, and then connected to a swivel contact that enables the animals to move freely. EEG signals were fed into a multi-channel differential amplifier, amplified (5000), band-pass (1–500 Hz) and notch filtered (50 Hz). The stimulation leads were connected to a programmed stimulator. The signal output was sampled at 512 Hz and digitized using a WINDAQ/Pro data acquisition system in combination with a DI410-interface (DATAQ Instruments 2.49, Akron, OH). Video was captured with a camera placed in the chamber and recorded with the aid of the Observer® (Noldus Information Technology BV, Wageningen, the Netherlands).

The animals had a 12:12 light/dark cycle with light at 8 A.M. because it was found that seizure occurrence was higher during the light than during the dark period (Raedt et al. 2009). The recording took place in a noise-isolated experimental chamber. Two days before EEG recording, the animals were placed in a Plexiglas recording cage (30 × 25 cm, high 35 cm) so as to habituate to the recording system. The rats were randomly assigned into two groups: a stimulation group (*n* = 10) where the rats received HFS and a sham group (*n* = 10) where the rats were connected with the stimulator but did not receive HFS. All rats received KA injections to induce seizures.

### Microinjection of KA

After 1-h baseline EEG recording, the animals received an injection of 0.05 μg KA (Ascent Scientific Ltd, Bristol, U.K.) and then were monitored with EEG and video of behavior for 1.5 h. KA injections were repeated every 1.5 h until the rats reached Stage V, Racine's scale (Racine 1972), that is, animals displayed convulsive seizures (bilateral myoclonus, tonic-myoclonus, rearing, and falling). The number of injections within 1 day was restricted to four. KA injections were conducted every other day for 3 days in total. When the animals showed convulsive SE for more than half an hour, diazepam was administered (5 mg/kg, i.p.) to control seizure severity.

During each injection, an injection needle was connected via tube to a 1 μL Hamilton syringe (Bonaduz, Switzerland) and was lowered into the guide cannula. The length of the injection needle was predetermined and the distance between the needle tip and the cannula tip was 0.5 mm. An amount of 0.1 μL KA (0.05 μg/0.1 μL; 0.1 μL/min) was injected from the syringe and then the needle was left in the cannula for another minute. Afterward, the needle was removed and a dummy was used to close the cannula.

### HFS

**Figure 1:**
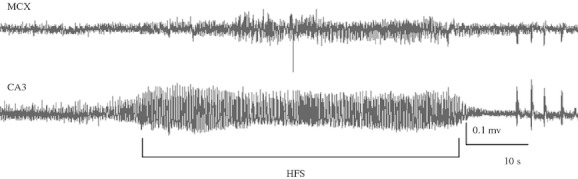
An example of seizure event on EEG. Epileptiform activity started on the CA3 channel with increasing amplitudes and frequency, and developed into high voltage spikes. Epileptic activity also occurred on the motorcortex channel with a delay. HFS was given to the subiculum area at the beginning of the seizure (300 dpi).

HFS was delivered at 125 Hz, bipolar, biphasic, square wave with a width of 100 μsec. The stimulation intensity was determined for each rat before the first KA injection. Starting with 100 μA, the intensity was step-wisely increased by 100 μA until motor effects (twitching, head nodding, rearing etc.) or EEG abnormalities were observed. Then the intensity was reduced by 200 μA and was kept at that level for the rest of the experiment. HFS parameters and the protocol to determine stimulation intensity were similar to what was used in previous studies (Velasco et al. 2001a,b, 2001; Vonck et al. 2002). HFS was triggered when epileptic activities on EEG were identified by visual inspection (interictal spikes increased with frequency and the amplitude surpassed twice the baseline EEG) without using any seizure detection algorithm and HFS lasted until EEG came back to the normal level (Fig. 1). The animals were continuously stimulated during seizures as well as during SE periods until the recording session was over. On average the delay between seizure start point and start of stimulation was 4.1 ± 0.3 sec (mean ± SEM).

### Histology

At the end of the experiment, the animals were anesthetized with sodium pentobarbital (60 mg/kg, i.p.) and later a DC current (25 μA, 15 sec) was delivered through the electrodes to create a lesion around the electrode tips. Afterward, the animals were perfused transcardially with 2% potassium ferrocyanide in a solution of 4% formaldehyde in 0.04 mol/L phosphate buffer (pH = 7.3). The brains were removed and post-fixed in the same solution overnight at 4°C. After post-fixation, the brains were placed into a 30% sucrose solution and remained there until they sank 3 or 4 days later. Then coronal sections (50 μm) were cut by a microtome (HM 440E; Microm, Waldorf, Germany) and the slices containing the track of the cannula and electrodes were stained with cresyl violet. In the end, these slices were examined under a light microscope to verify the positions of the cannula and electrodes using the atlas of the rat brain (Paxinos and Watson 1998).

### Data analysis

The recorded EEG was reviewed with the WINDAQ/Pro browser. The start point of a seizure was defined when the amplitude of the spikes in a spike train was twice the baseline on EEG. Racine (1972) score was used to classify the severity of behavioral seizures: Stage I (immobility, facial automatism), II (head nodding, wet dog shakes), III (unilateral myoclonus), IV (bilateral myoclonus or tonic-myoclonic behavior, rearing without falling) and V (bilateral myoclonous or tonic-myoclonic behavior, rearing and falling). Focal seizures were defined both by severity of seizure behavior (Stage I or II) and electrographic seizures on CA3 channel. Generalized seizures were defined both by seizure severity (Stage III, IV, or V) and electrographic seizures that synchronized on both local CA3 channel and motorcortex channel on EEG. Status epilepticus (SE) was defined as continuous seizures on EEG for more than 30 min. Discrete convulsive seizures occurred before the presence of SE. Seizure characteristics such as seizure number, latency, duration, and inter-seizure interval were calculated, and seizure severity was scored. Interictal spikes (IS) were counted as IS rate (IS number/min) in the session (90 min) in which the first seizure occurred. IS were detected with an offline custom made spike detection program and the IS rate was calculated on all 3 days. Statistical analysis was done with the aid of SPSS 15.0 (IBM Corporation, Somers, New York). Repeated-measures ANOVA was used to examine day and group effects on seizure parameters. If appropriate, post-hoc independent and dependent Student's *t*-tests were further used.

### Algorithm for IS detection

An offline spike detection algorithm was used only to detect IS. A spike on EEG was distinguished from the background activity with a pointed peak, amplitude at least twice the background activity and duration from 20 to 70 msec (Chatrian et al. 1974). Features of the normal background EEG such as mean (μ) and variance (σ^2^) of amplitudes were first extracted from the baseline EEG. The amplitude of the normal background EEG is considered to have a Gaussian distribution. For any given datapoint of signal in the subsequent recording after KA injection, the probability of its amplitude was computed as


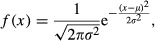


where *f*(*x*) is a probability density function for each datapoint *x*, μ is the mean amplitude and σ^2^ is the variance of the amplitude of the baseline EEG. When the variance of a given datapoint is higher than a certain cutoff threshold, it is considered as a spike.

The cutoff threshold (*T*) in the algorithm is defined as


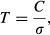


where *C* represents a constant value that was empirically chosen. A range of *C* values were explored to find a proper threshold for each rat individually to obtain a mean specificity and sensitivity higher than 85% in spike detection (based on randomly selected piece of data for each rat, see one example in Fig. S1).

## Results

Histology results showed that the stimulation electrodes were located outside the subiculum in five rats and therefore these rats were excluded from this study. For the rest of rats, the cannula was located in the CA3 area of the hippocampus and the stimulation electrode was placed in the subiculum (Fig. S2). In total, 15 rats were included for data analysis in the stimulation (*n* = 7) and sham group (*n* = 8).

Epileptic activities were induced after KA injection. It started with low-amplitude fast activities, increased gradually with amplitude and frequency, and finally developed into full-blown spike trains on the hippocampal channel, with or without generalization of the motor cortex channel (Fig. 1).

Table 1 gives an overview of KA injections that the animals received on 3 days. On the first day, four rats of both groups reached SE. The number of injections did not differ in these two groups, nor did the SE percentage that day. On the second day, one rat of the HFS group did not develop convulsive seizures while two of the sham group did not display convulsive seizures. On the third day, two rats of both groups did not show generalized seizures.

**Table 1:** Severity of seizures after each injection (max. four) over 3 days

	Day 1				Day 2				Day 3			
			
Sham (*n* = 8)	1	2	3	4	1	2	3	4	1	2	3	4
1	V*				V				I	I	I	I
2	II	V			V				I	V		
3	II	V*			I	II	II	I	I	I	I	I
4	V				V				V			
5	V*				I	I	I	I	V*			
6	V				V				V			
7	V				V				V*			
8	V*				I	V			I	I	II	V

	Day 1				Day 2				Day 3			
			
HFS (*n* = 7)	1	2	3	4	1	2	3	4	1	2	3	4
1	V				V*				V			
2	I	V			V				II	V		
3	I	V*			V*				V			
4	I	V*			I	V			I	I	I	II
5	V*				I	I	I	I	I	I	I	I
6	V				I	V			V			
7	V*				V				V			

Seizure severity: Stage I–V according to Racine scale.

* Animal reached SE and received diazepam injection.

**Figure 2:**
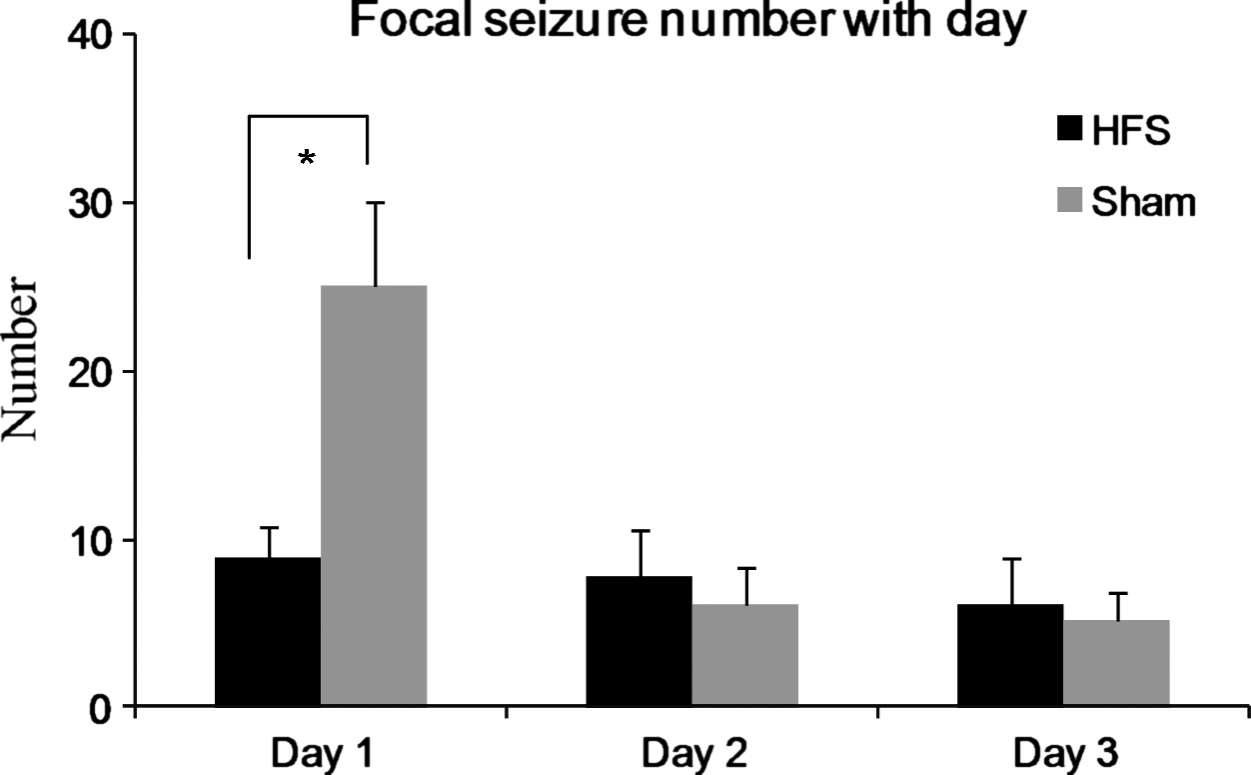
Represents the number of focal seizures (mean ± SEM) on 3 days in the HFS and sham groups. Note that the HFS group had less focal seizures on the first day. HFS, high frequency stimulation (300 dpi).

The parameters such as number, duration, and latency of focal and generalized seizures were calculated for the three injection days (Table 2). For focal seizure number, a day effect (*F*_(2,26)_ = 11.50, *P* < 0.01) and interaction effect between day and group (*F*_(2,26)_ = 7.63, *P* < 0.01) were found. The number of focal seizures was 8.9 ± 1.7 in the HFS group and 25.1 ± 5.1 (mean ± SEM) in the sham group on Day 1: this difference was significant (*t*_(13)_ = 2.84, *P* < 0.05) (Fig. 2). In addition, the number of focal seizures, independent of group, was less on Day 2 (*t*_(14)_ = 2.90, *P* < 0.05) and Day 3 (*t*_(14)_ = 3.23, *P* < 0.01) compared to Day 1.

**Table 2:** Number, duration, and latency of focal and generalized seizures in the HFS and sham group

	Focal seizure number, mean ± SEM			Generalized seizure number, mean ± SEM		
		
	Day 1	Day 2	Day 3	Day 1	Day 2	Day 3
HFS (*n* = 7)	8.9 ± 1.7^*^	7.8 ± 2.8	6.2 ± 2.8	2.4 ± 0.6	2.3 ± 0.6	2.5 ± 1.0
Sham (*n* = 8)	25.1 ± 5.1	6.1 ± 2.3	5.2 ± 1.7	1.7 ± 0.3	1.6 ± 0.5	2.7 ± 0.8

	Focal seizure duration (sec), mean ± SEM			Generalized seizure duration (sec), mean ± SEM)		
		
	Day 1	Day 2	Day 3	Day 1	Day 2	Day 3
HFS (*n* = 7)	36.3 ± 5.9	23.7 ± 7.1	14.0 ± 5.3	83.4 ± 13.7	43.5 ± 12.3	29.5 ± 8.6
Sham (*n* = 8)	35.0 ± 4.0	22.2 ± 4.7	22.3 ± 6.1	107.8 ± 11.3	45.0 ± 12.7	38.2 ± 11.7

	Focal seizure latency (min), mean ± SEM			Generalized seizure latency (min), mean ± SEM		
		
	Day 1	Day 2	Day 3	Day 1	Day 2	Day 3
HFS (*n* = 7)	27.1 ± 7.7	45.1 ± 9.7	54.1 ± 11.8	43.3 ± 10.7	57.3 ± 7.4	69.8 ± 7.9
Sham (*n* = 8)	10.6 ± 3.4	39.2 ± 12.6	46.5 ± 13.4	51.1 ± 11.2	56.4 ± 13.2	51.0 ± 12.9

HFS, high frequency stimulation. Note that the HFS group had a significant reduction of focal seizure number (marked by ^*^) on Day 1.

A logarithmic transformation was applied to the inter-seizure interval as it was not normally distributed. The inter-focal seizure interval (log) was 2.4 ± 0.1 sec in the HFS group and 2.0 ± 0.1 sec in the sham group on Day 1. Independent *t*-test showed that the HFS group had a longer inter-focal seizure interval (*t*_(13)_ = 2.38, *P* < 0.05) than the sham group. Considering the low number of seizures and that a few animals were no longer responsive to KA injections on Day 2 and 3, no further tests were done.

**Figure 3:**
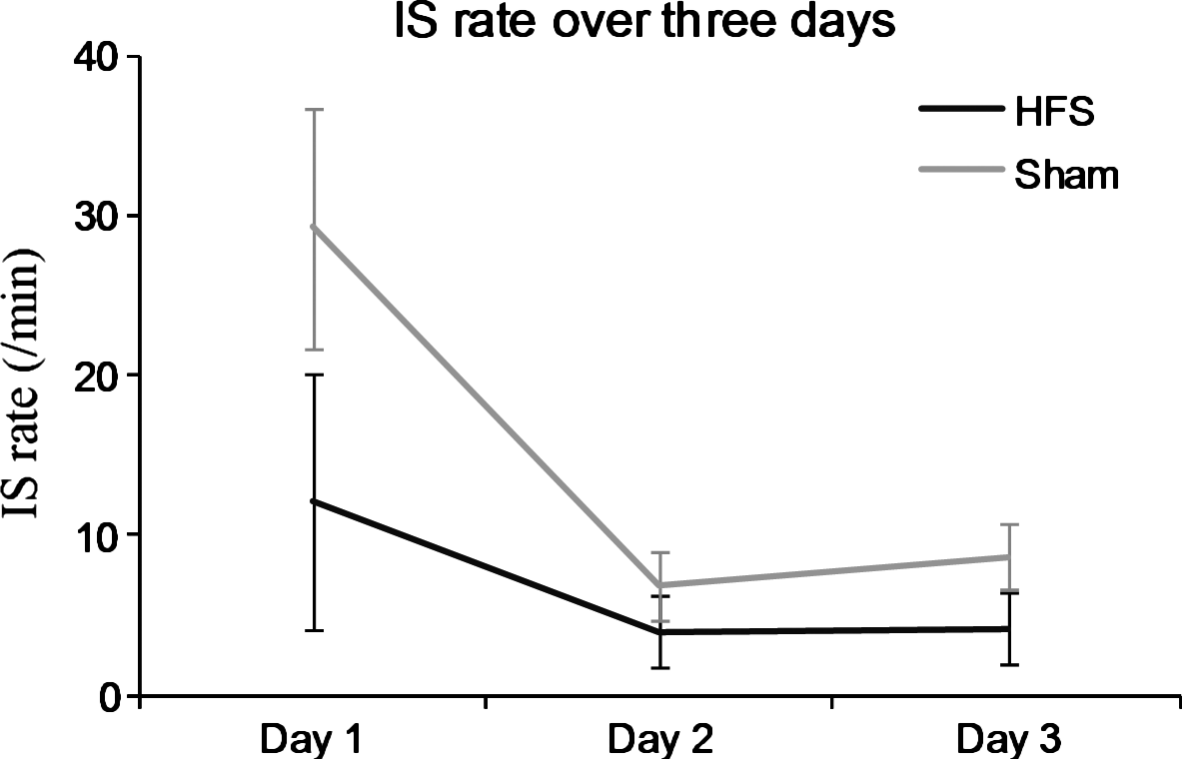
Illustrates the interictal spike (IS) rate (number/min) (mean ± SEM) over after the first seizure occurrence over 3 days. Note that the IS rate was lower in the HFS group and it also decreased over injection days. The IS rate was defined as number of spikes per minute (300 dpi).

The IS rate was calculated on 3 days for the two groups (Fig. 3). An overall group effect was found for the IS rate: it was lower in the HFS group (*F*_(1,13)_ = 5.54, *P* < 0.05) than in the sham group. A day effect was also found (*F*_(2,26)_ = 5.67, *P* < 0.01) for the IS rate. Post-hoc comparisons revealed that the IS rate on Day 1 was higher than on Day 2 (*P* < 0.05) and Day 3 (*P* < 0.05).

Significant day effects were also found for the latency (*F*_(2,26)_ = 6.94, *P* < 0.01), duration of focal seizures (*F*_(2,26)_ = 5.65, *P* < 0.01) and duration of generalized seizures (*F*_(2,26)_ = 19.41, *P* < 0.01). Post-hoc *t*-test showed less duration of focal (*t*_(14)_ = 2.27, *P* < 0.05) and generalized seizures (*t*_(14)_ = 4.11, *P* < 0.01), and longer latency of focal seizures (*t*_(14)_ = 2.95, *P* < 0.05) on Day 2 compared to Day 1. Similarly, the duration of focal seizures (*t*_(14)_ = 2.91, *P* < 0.05) and generalized seizures (*t*_(14)_ = 5.38, *P* < 0.01) were shorter, together with increased focal seizure latency (*t*_(14)_ = 3.65, *P* < 0.01) on Day 3 compared to Day 1.

## Discussion

Acute responsive HFS was applied to the subiculum on KA induced seizures in rats. The major outcomes were that: (1) Acute responsive HFS did not disrupt or shorten ongoing electrographic seizures. (2) The HFS group had less focal seizures and longer inter-seizure interval after the first seizure on Day 1. Lower IS rate was also found in the HFS group. (3) Sensitivity to KA injections decreased over injection days for both groups.

First of all, responsive HFS did not disrupt or shorten ongoing seizures in this KA induced seizure model. This result is in contrast with the effects of HFS on absence seizures. Two studies (Sarkisian et al. 1997; Vercueil et al. 1998) showed that bilateral HFS (130 Hz) of the subthalamic nucleus interrupted ongoing absence seizures. The discrepancy in the effects of responsive HFS in these two types of seizures are not surprising considering that temporal lobe epilepsy and absence epilepsy involve various neuronal networks, manifested with different etiologies, clinical profiles, treatment strategies, and intensity—absences are characterized by mild seizures. On the other side, some in-vitro studies suggested that application of high frequency stimulation (100 Hz) or electric field (50 Hz sinusoid field) to the hippocampal slices could curtail the epileptiform activities such as interictal-like activities (Bikson et al. 2001) or ictal activities induced by low calcium, picrotoxin or high potassium (Lian et al. 2003; Su et al. 2008). However, to the best of our knowledge, no in-vivo study has reported a complete abortion of seizure activities by acute stimulation in temporal lobe seizure or epilepsy models.

Meanwhile, our results suggest that responsive HFS of the subiculum had anticonvulsant effects on the first day in terms of less focal seizures and a longer inter-focal seizure interval. These results are in line with the preliminary findings in Nair et al. (2006) study in which decreased seizure frequency and increased free seizure period were found after acute hippocampal stimulation in the SE model. The subiculum is known as the principal output structure for the hippocampus formation. One feature of the subiculum is the presence of bursting cells that fire bursts of action potentials in response to single orthodromic stimulation (Stewart and Wong 1993). Moreover, like the CA3 area, the subiculum possesses a certain density of recurrent excitatory connections, which are crucial for generation of synchronized activity (Heinemann 1987). Such intrinsic cellular and network properties of the subiculum render it a seizure-prone area. Neurophysiological evidence in human and experimental animal models further support the hyperexcitability of the subiculum. Spontaneous rhythmic activities were found in the subiculum in brain slices of TLE patients with or without hippocampal sclerosis (Cohen et al. 2002; Wozny et al. 2003), resembling the epileptiformic activities observed in EEG of TLE patients. Similar interictal or ictal-like activities were also generated in the isolated subiculum in in-vitro rat models of TLE (Behr and Heinemann 1996; Menendez de la Prida and Gal 2004). Taken together, the intrinsic properties of the subiculum and evidence on electrophysiological studies favor the hypothesis that the subiculum is prone to synchronous activity and involved in seizure generation.

It is necessary to point out that some small anatomical and physiological circuits were found such as presubiculum-subiculum (Funahashi et al. 1999) and subiculum-CA1 (Harris and Stewart 2001) as a result of re-entrant activity. These small regional circuits facilitate synchronization of various areas within the hippocampal network and thus amply seizure activities. The stimulation of the subiculum can activate these re-entrant pathways to further act on their downstream structures, therefore influence seizure initiation.

Meanwhile, interictal spikes were also suppressed by responsive HFS. Previous studies in patients also showed that IS were reduced by HFS (Velasco et al. 2000a; Boex et al. 2007). Despite it remains questionable whether the rate of IS is a valid measurement of epileptogenic activities (Gotman and Marciani 1985; Katz et al. 1991), the presence of IS is believed to be highly associated with epilepsy and they could indicate the occurrence of upcoming ictal events. IS rate is also used as an important criterion to assess the efficacy of DBS in acute stimulation in TLE patients (Boon et al. 2007). Although the exact mechanism remains largely unknown, IS are thought to represent the extracellular synchronous and excessive discharge of neuronal ensembles (Nakagawa and Durand 1991; Warren and Durand 1998). It is therefore assumed that HFS suppresses this synchronous discharges.

It is also noted that the effects of acute responsive HFS on focal seizure were found only on Day 1, indicating that HFS reduced excitability of local network temporarily. A study of chronic stimulation (Wyckhuys et al. 2010b) has also found that seizure frequency returned to baseline level after termination of Poisson distributed HFS. On the other hand, around half of the rats reached SE at the end of recording on Day 1 and received injection of diazepam to control seizure severity. Thus, it is also possible that occurrence of SE, injection of diazepam or both might be confounding factors, masking, if any, the effect of acute responsive HFS on the subsequent days.

The last, a reduction of sensitivity to KA injection was found over injection days. Such tolerance to the effects of KA was reported on the developing brain of rats (Holmes and Thompson 1988; Sarkisian et al. 1997). Previous studies have shown that different stimuli including KA, kindling, and spontaneous seizures can precondition the brain and may protect against subsequent seizures (Kelly and McIntyre 1994; Sasahira et al. 1995; Najm et al. 1998; Plamondon et al. 1999). Severe epileptic conditions such as SE could provoke an intrinsic anticonvulsant mechanism, resulting in seizure insensitivity to subsequent injections when given 48 h later.

## Conclusion

Responsive HFS of the subiculum suppressed focal seizures and IS without interrupting ongoing seizures in this acute seizure model. Such anticonvulsant effects of acute subicular stimulation indicate that the subiculum is involved in seizure generation. The reduction of seizure sensitivity over the injection days might reflect an intrinsic anticonvulsant mechanism.

## Supporting Information

**Figure S1.** An example of detection of interictal spikes (IS). The upper channel represents a piece of EEG data containing some IS on the CA3 channel. The lower channel represents IS detection by an offline IS detection algorithm. Each vertical line represents positive detection of one interical spike. The movement artifact (marked by arrow) is not detected as an interictal spike (300 dpi).

**Figure S2.** An example of the locations of one cannula and stimulation electrode in one slice. The white arrow refers to the cannula tip in the CA3 area and the black arrow shows the stimulation electrode tip in the subiculum of the hippocampus. S, subiculum (300 dpi).
