# Supplementary material for: The effects of acute responsive high frequency stimulation of the subiculum on the intra-hippocampal kainic acid seizure model in rats
Source: Brain Behav. 2012 Jul 10;2(5):532–40. doi: 10.1002/brb3.70 (PMC3489806; doi:10.1002/brb3.70)
Supplement: Supplementary file 1 [file brb30002-0532-SD1.doc]

Supplemental Figure 1


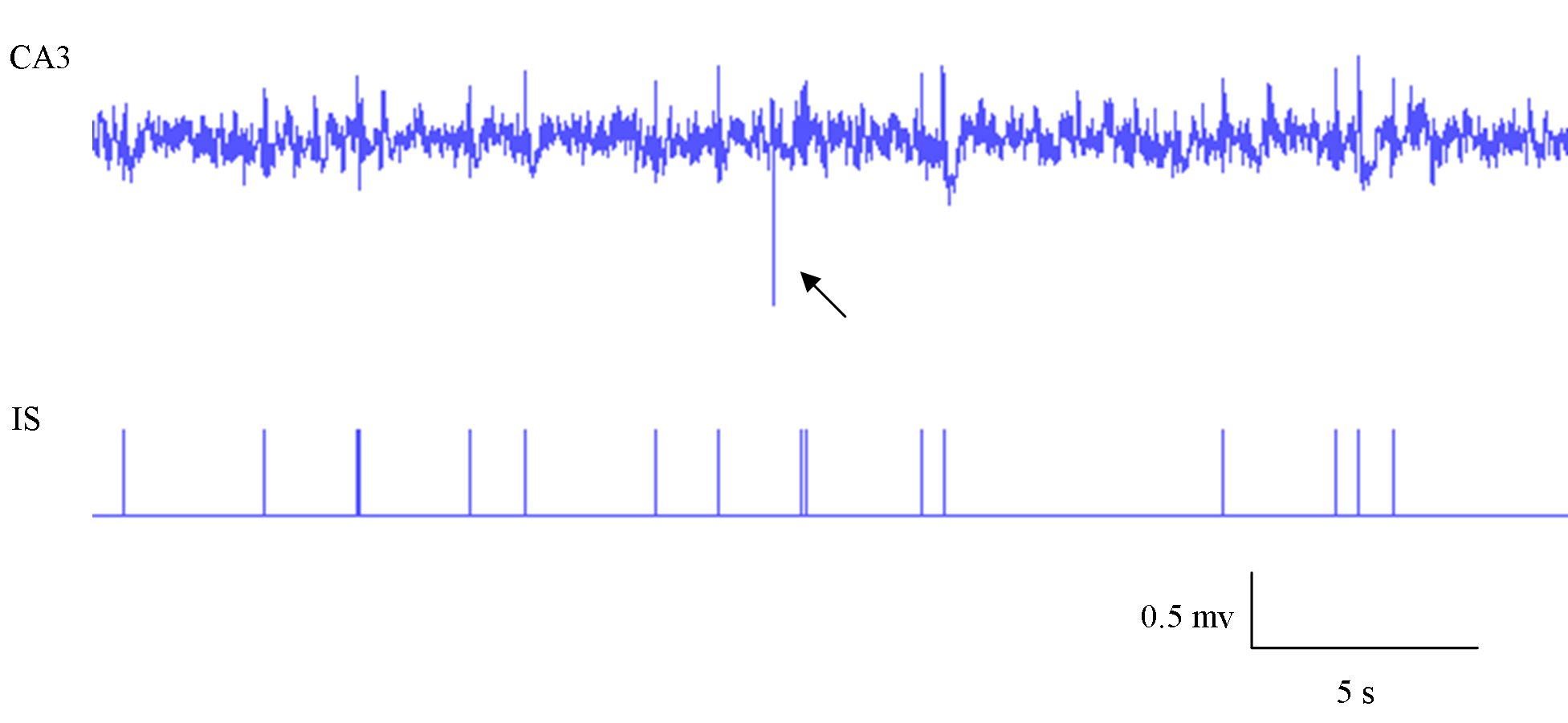


Fig. 1 illustrates an example of detection of interictal spikes (IS). The upper channel represents a piece of EEG data containing some IS on the CA3 channel. The lower channel represents IS detection by an offline IS detection algorithm. Each vertical line represents positive detection of one interical spike. The movement artifact (marked by arrow) is not detected as an interictal spike (300 dpi)

Supplemental Figure 2


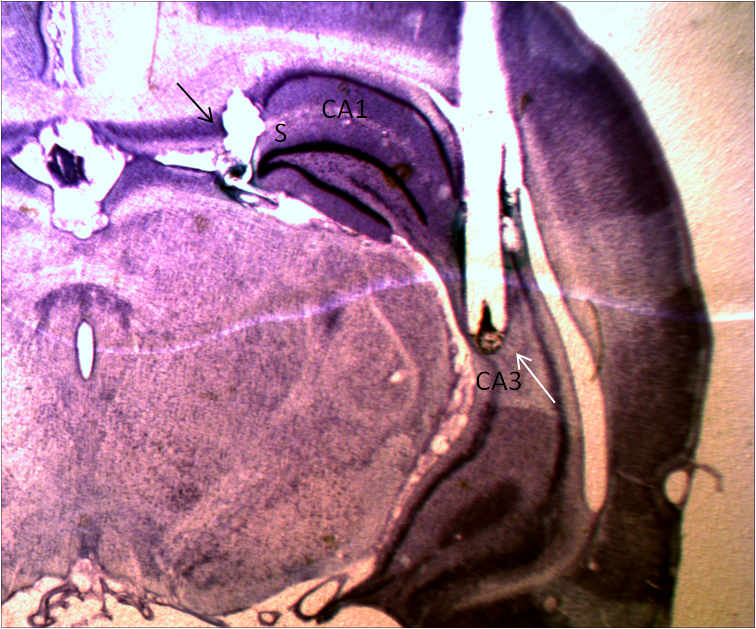


Fig. 2 illustrates an example of the locations of one cannula and stimulation electrode in one slice. The white arrow refers to the cannula tip in the CA3 area and the black arrow shows the stimulation electrode tip in the subiculum of the hippocampus. S: subiculum. (300 dpi)
